# Extent of Single-Neuron Activity Modulation by Hippocampal Interictal Discharges Predicts Declarative Memory Disruption in Humans

**DOI:** 10.1523/JNEUROSCI.1380-19.2019

**Published:** 2020-01-15

**Authors:** Chrystal M. Reed, Clayton P. Mosher, Nand Chandravadia, Jeffrey M. Chung, Adam N. Mamelak, Ueli Rutishauser

**Affiliations:** ^1^Department of Neurology,; ^2^Department of Neurosurgery,; ^3^Center for Neural Science and Medicine, Department of Biomedical Sciences, Cedars-Sinai Medical Center, Los Angeles, California 90048, and; ^4^Division of Biology and Biological Engineering, California Institute of Technology, Pasadena, California 91125

**Keywords:** declarative memory, episodic memory, hippocampus, human-single neuron, interictal epileptic discharges, intracranial

## Abstract

Memory deficits are common in epilepsy patients. In these patients, the interictal EEG commonly shows interictal epileptiform discharges (IEDs). While IEDs are associated with transient cognitive impairments, it remains poorly understood why this is. We investigated the effects of human (male and female) hippocampal IEDs on single-neuron activity during a memory task in patients with medically refractory epilepsy undergoing depth electrode monitoring. We quantified the effects of hippocampal IEDs on single-neuron activity and the impact of this modulation on subjectively declared memory strength. Across all recorded neurons, the activity of 50 of 728 neurons were significantly modulated by IEDs, with the strongest modulation in the medial temporal lobe (33 of 416) and in particular the right hippocampus (12 of 58). Putative inhibitory neurons, as identified by their extracellular signature, were more likely to be modulated by IEDs than putative excitatory neurons (19 of 157 vs 31 of 571). Behaviorally, the occurrence of hippocampal IEDs was accompanied by a disruption of recognition of familiar images only if they occurred up to 2 s before stimulus onset. In contrast, IEDs did not impair encoding or recognition of novel images, indicating high temporal and task specificity of the effects of IEDs. The degree of modulation of individual neurons by an IED correlated with the declared confidence of a retrieval trial, with higher firing rates indicative of reduced confidence. Together, these data link the transient modulation of individual neurons by IEDs to specific declarative memory deficits in specific cell types, thereby revealing a mechanism by which IEDs disrupt medial temporal lobe-dependent declarative memory retrieval processes.

**SIGNIFICANCE STATEMENT** Interictal epileptiform discharges (IEDs) are thought to be a cause of memory deficits in chronic epilepsy patients, but the underlying mechanisms are not understood. Utilizing single-neuron recordings in epilepsy patients, we found that hippocampal IEDs transiently change firing of hippocampal neurons and disrupted selectively the retrieval, but not encoding, of declarative memories. The extent of the modulation of the individual firing of hippocampal neurons by an IED predicted the extent of reduction of subjective retrieval confidence. Together, these data reveal a specific kind of transient cognitive impairment caused by IEDs and link this impairment to the modulation of the activity of individual neurons. Understanding the mechanisms by which IEDs impact memory is critical for understanding memory impairments in epilepsy patients.

## Introduction

Cognitive deficits are common in chronic epilepsy patients. The exact mechanism underlying these deficits is unclear and may be due to structural damage, ongoing abnormal electrical activation, medication side effects, or a combination of these processes. Interictal epileptiform discharges (IEDs) are brief high-amplitude pathological discharges commonly seen in between seizures in some epilepsy patients (de Curtis et al., 1999; de Curtis and Avanzini, 2001; Cohen et al., 2002). These discharges typically occur within or around the seizure onset zone. Although IEDs are typically considered to be asymptomatic, there is some evidence that they are related to brief lapses in cognition (Aarts et al., 1984; Aldenkamp and Arends, 2004; Aldenkamp et al., 2004; Horak et al., 2017; Ung et al., 2017).

Most prior work on the relationship between epileptic IEDs and cognition has been performed using scalp EEG (Schwab, 1939; Rausch et al., 1978; Aarts et al., 1984). Because the extent to which IEDs originating from the hippocampus and other deep structures can be captured using scalp EEG is limited, it remains unclear how hippocampal memory processes are modulated by IEDs. More recently, work using intracranial EEG (implanted depth or subdural grid electrodes) in epilepsy patients has started to reveal a better understanding of the relationship between neural activity, cognitive processes, and their impairment by IEDs (Kleen et al., 2013; Horak et al., 2017; Ung et al., 2017). Several studies have found that the occurrence of IEDs recorded with intracranial electrodes correlates with impaired behavioral performance in working memory (Krauss et al., 1997; Ung et al., 2017) and delayed free recall tasks (Kleen et al., 2013; Horak et al., 2017). Moreover, it was found that IEDs outside a left-hemispheric seizure onset zone impacted memory encoding, recall, and retrieval, while those inside the seizure onset zone did not (Ung et al., 2017). While these studies reveal correlations between the occurrence of IEDs and behavioral effects, it remains unknown why IEDs are indicative of such impairment and what specific neuronal processes they disrupt. In particular, the temporal specificity between the occurrence of an IED and the disruption of the observed memory deficits is unclear.

IEDs are thought to be the result of large synchronous bursts of neuronal activity. In humans, this view is supported by a small number of pioneering single-neuron studies that have revealed that a subset of up to ∼30% of neurons increase or decrease their firing transiently prior or during an IED (Alarcón et al., 2012; Alvarado-Rojas et al., 2013). The sparse and highly variable involvement of ∼30%-40% of neurons during an IED makes it difficult to study the exact role of such neuronal modulation in this abnormal network activity. While these studies reveal prominent modulation of single-neuron activity by IEDs, it remains unknown whether such modulation is detrimental to memory performance or whether, alternatively, the neurons engaged in a particular task are not influenced by IEDs.

We used hybrid depth electrodes in human epilepsy patients to study the relationship between single neuron activity and hippocampal IEDs during a hippocampal memory-dependent new/old recognition memory task that is frequently used to study aspects of human declarative memory. In this task, subjects were first shown a series of novel images (“encoding”). Later, subjects were again shown the same images randomly intermixed with novel images not seen before (“retrieval”). During retrieval, patients were asked to indicate whether a displayed image was new or old, and how confident they were in their decision. This allowed us to study the effects of IEDs during both encoding and retrieval. This task has been widely studied in humans using a variety of techniques, including scalp EEG, single-neuron activity, and fMRI (Rugg and Curran, 2007; Guerin and Miller, 2009; Fried et al., 2014), making it well suited to study the effects of hippocampal IEDs in patients with medically refractory epilepsy undergoing depth electrode invasive intracranial monitoring to localize seizures.

## Materials and Methods

### 

#### Subjects

Nineteen patients (Table 1) with intractable epilepsy underwent depth electrode monitoring for localization of the seizure focus as part of their presurgical plan for resection. Of the 19 patients, we excluded 2 from analysis because they had no IEDs during the task and 5 because they had a seizure <1 h before, or after testing. In total 23 behavioral testing sessions were analyzed. One of the analyzed patients had 3 sessions of the task, and the rest had only 1 session. We also excluded 1 patient (P32) that only had generalized spike and wave discharges, leaving 11 patients (13 sessions) with hippocampal IEDs for the final analysis. The study was approved by the Cedars-Sinai Institutional Review Board (IRB 13369), and all patients provided written informed consent. Electrode localization was based on clinical criteria only.

**Table 1.** List of the 12 subjects analyzed*^a^*

Patient ID	Type of IEDs	Seizure onset zone
P32	Generalized spike and wave	Undetermined
P34	Left hippocampal	Bitemporal
P35	Left hippocampal	Left temporo-neocortical
P36	Right hippocampal	Right medial temporal
P38	Bitemporal	Right medial temporal
P39	Bitemporal	Right insular
P47	Bitemporal	Left medial temporal
P48	Bitemporal	Left neocortical
P49	Bitemporal	Left amygdala
P54 (x3)	Bitemporal and generalized spike and wave	Right medial temporal
P55	Right hippocampal	Right medial temporal
P56	Left hippocampal	Bitemporal

*^a^*Each subject contributed one session, except P54, who contributed 3 sessions.

#### Experimental design

##### Memory task.

**Figure 1.**
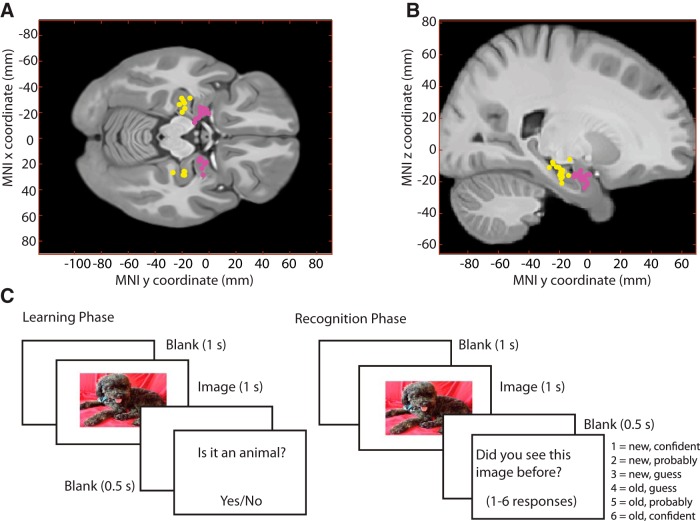
Electrode placement and the recognition memory task. Electrode locations across all patients, projected onto an (***A***) axial (*z* = −16) and (***B***) sagittal (*x* = 22.1) view. All electrode locations for which at least one usable electrode was recorded are shown. Yellow represents hippocampus. Pink represents amygdala. ***C***, The task is composed of a learning phase, during which 100 new images are shown to the subjects. During the recognition test phase, they are shown both new and old images and have to report whether they have seen each image before by reporting a new/old decision together with a confidence level on a 1–6 scale.

The task used has been previously described (Rutishauser et al., 2015; Faraut et al., 2018). There are three versions of the task, which are all identical, except for the images shown. Each stimulus set contains images chosen from five different visual categories (cars, food, people, landscape, animals), with an equal number of instances chosen from each. The experiment consisted of two parts: a learning block and a recognition block (Fig. 1*C*). During the learning block, subjects were shown 100 new images. Each image was only shown once for 1 s. During the recognition block, a random subset of 50 of these images was shown again (old) randomly mixed with a set of 50 new images. After each image, subjects were asked whether they had seen this identical image before (old) or not (new) and with what confidence. Subjects provided their answer on a 1–6 confidence scale as follows: 1 = new, very sure; 2 = new, sure; 3 = new, guess; 4 = old, guess; 5 = old, sure; 6 = old, very sure. Patients provided their answers by pressing buttons on an external response box (RB-740, Cedrus). The task was implemented in MATLAB using the Psychophysics toolbox.

##### Electrode and data acquisition.

All recordings were performed with hybrid (macro-micro) depth electrodes (BF08R-SP05X-000 Behnke-Fried and WB09R-SP00X-0B6, AdTech Medical). Each electrode contained an inner bundle of eight 40-μm-diameter microwires that protruded 4–5 mm from the distal end of the clinical electrode and could record single neuron extracellular action potentials (single units) (Fried et al., 1999; Minxha et al., 2018). The signal from each microwire was locally referenced to 1 of the 8 microwires, thus allowing the recording of activity from 7 microwires in each area. Data were recorded broadband (0.1–9000 Hz filter) sampled at 32 kHz using either an Atlas or Cheetah (Neuralynx) system.

All patients were implanted in the hippocampus, amygdala, presupplementary motor area (pre-SMA), anterior cingulate cortex (ACC), and orbitofrontal cortex. Throughout the manuscript, medial temporal lobe (MTL) refers to amygdala and hippocampus together. Similarly, we refer to all cortical recording sites together as medial frontal cortex. One patient was implanted with additional electrodes in the insular cortex, and 1 had additional electrodes placed in the lateral anterior temporal neocortical areas identified as a possible epileptogenic zone with MEG. We only performed single-neuron recordings from amygdala, hippocampus, dorsal ACC, pre-SMA, and orbitofrontal cortex; thus, our focus here is only on these brain areas.

#### Statistical analysis

##### Action potential (“spike detection”) and sorting.

For each channel, the raw signal was bandpass filtered 300–3000 Hz. Activity was sorted to identify putative individual neurons using the semiautomatic template-matching algorithm OSort, which is available as open source (Rutishauser et al., 2006a). This method has been described in detail previously (Faraut et al., 2018).

##### IEDs.

**Figure 2.**
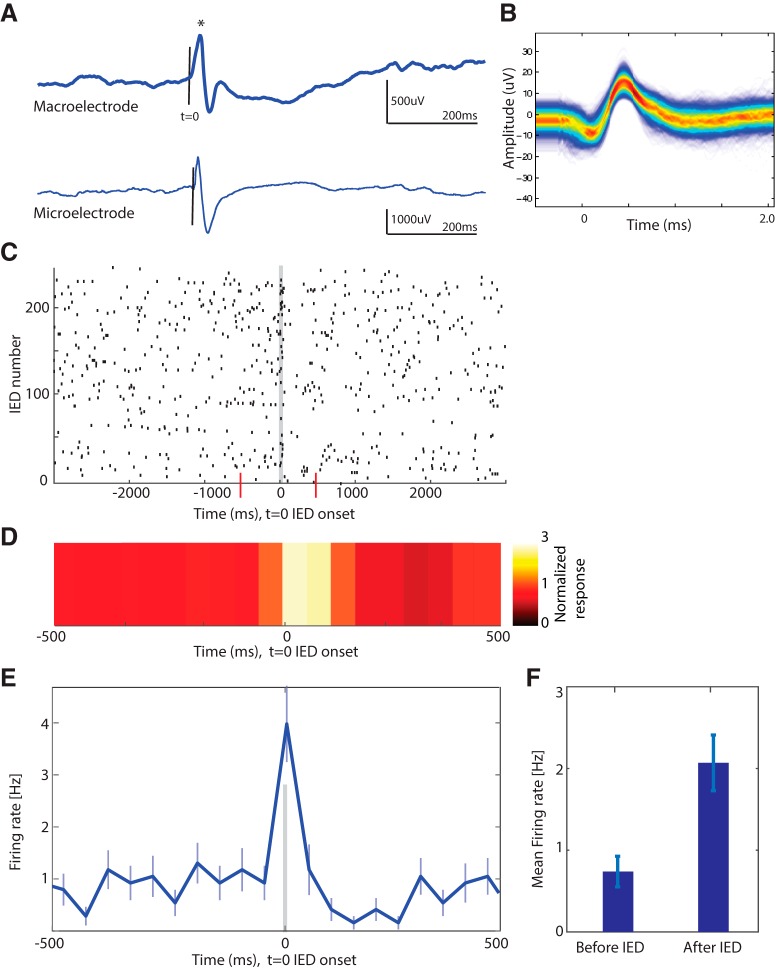
Relationship between IEDs in the intracranial EEG and single-neuron activity. ***A***, Example IED. Shown is the raw iEEG recording from a simultaneously recorded right hippocampal macroelectrode (top) and microelectrode (bottom) of p48. *Peak of the IED. ***B***, The waveform of the action potential of a modulated unit recorded from the same microwire as shown in ***A***. ***C***, Raster plot of the unit shown in ***B***, aligned to the IED onset at *t* = 0. Each row represents a different IED. Red lines indicate the ±500 ms around the IED. ***D***, Heatmap of the average firing rate of the neuron shown in ***B*** and ***C*** in a window ±500 ms around the IED. Each data point indicates the mean firing rate in a 25 ms bin. Scale of the normalized response shown on right, with the color representing the change in firing rate from baseline (e.g., a value of 3 indicates the firing rate is 3 times higher than baseline). ***E***, PSTH of the data shown in ***C*** in a window ±500 ms around the IED. Each data point indicates the mean firing rate in 50 ms bin. Error bars indicate SEM of the mean firing rate. There are different time scales in ***C***, ***D***, ***E***. ***F***, The mean firing rate for the unit shown in ***B–E*** shows an ∼100% increase in firing of the unit during the IED relative to baseline. Error bar indicates SEM of the mean firing rate.

Given the poor interrater reliability of automatic IED detection (Gaspard et al., 2014), we used visual inspection of the macro and micro channels to detect IEDs. Each identified IED was manually validated by a board certified epileptologist (C.M.R.). Discharges on hippocampal microelectrode and macroelectrode recording showing a biphasic or triphasic morphology with an initial fast phase of ≤200 ms were chosen (see Fig. 2*A*). These discharges may or may not have been followed by an after-going slow wave. Time 0 was defined as the first change from the baseline of the fast component (see Fig. 2*A*, vertical line). Others sometimes use the peak of the fast component as time 0 (Keller et al., 2010). Recordings were bilateral, and we marked right and left IEDs independently. Thus, in the few patients that had hippocampal IEDs occurring bilaterally, not simultaneously, we designated these as separate events. For the purpose of this study, we identified IEDs only on the hippocampal contacts. However, we found that ∼99% of these IEDs were also visible on the amygdala microelectrode contacts in the amygdala and could thus be designated as medial temporal IEDs. However, given that the time stamps were generated from the hippocampal microelectrode contact, we refer to them here as hippocampal IEDs throughout.

One patient had both independent hippocampal and generalized spike and wave discharges. For this patient, the generalized and hippocampal IEDs were marked separately. IEDs were selected during the entire new/old task on the microelectrode recording and confirmed with the macroelectrode recording. Since we wanted to avoid peri-ictal or ictal-related discharges (Gotman and Koffler, 1989; Karoly et al., 2016), we eliminated sessions in which an ictal event occurred <1 h before start of the task. IEDs were inspected and marked in EEGLAB with the VisEd plugin (Delorme and Makeig, 2004). The median rate of IEDs across all subjects was 0.0863 per second (0.007–0.442/s, SD ± 0.1419).

#### Electrode localization

For each patient, the microelectrode positions were localized from MRI scans performed after implantation of electrodes. These scans were registered to preoperative MRI scans using Freesurfer's MRI_robust_register as described previously (Faraut et al., 2018) (Fig. 1).

#### Data analysis of modulation of single-neuron firing by IEDs

We examined in total 728 isolated single units across 11 patients. To quantify the time course of IED-related modulation of single-neuron activity, time 0 (“start of the IED”) was identified as the first change from the baseline of the fast component of the IED, not the peak of the fast component as mentioned by Keller et al. (2010) (see Fig. 2*A*, asterisk). We defined a neuron to be modulated by an IED if the neurons firing rate during the 0–50 ms time period following the start of the IED was significantly different from that of the firing rate within 50 ms before the IED (−50 to 0 ms), evaluated using a two-tailed *t* test at *p* < 0.05. We further quantified the modulation of the activity of a neuron by an IED using a modulation index (MI), defined as MI = (mean firing rate after IED) − (mean firing rate before IED)/(mean firing rate after IED + mean firing rate before IED). Here, the mean firing rate was again quantified in 50 ms bins before/after *t* = 0 of IED onset. An MI of 0 indicated no modulation. A negative MI indicates a decrease in the neuronal firing rate due to the IED, and a positive MI indicates an increase in firing rate due to the IED. We also calculated Cohen's *d*, defined as follows: score = (mean firing rate after IED − mean firing rate before IED)/SD, to further characterize the strength of modulation. Here as above, the mean firing rate was quantified in 50 ms bins before/after *t* = 0 of IED onset.

**Figure 3.**
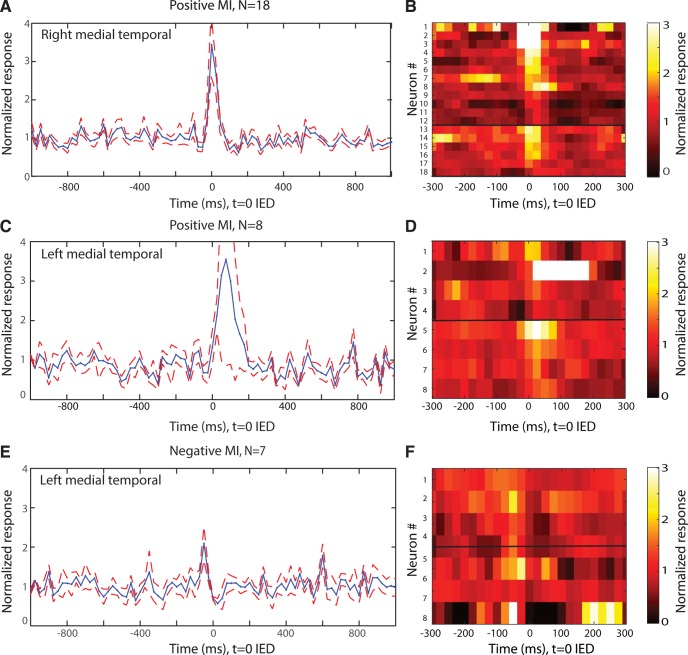
Time course of modulation of single-neuron activity by IEDs. PSTH of the modulation of neuronal activity averaged across all modulated neurons, split according to right (***A***,***B***) and left temporal region (***C–F***). ***A***, PSTH of all modulated neurons in the right MTL. All had positive MIs. ***B***, Heatmap showing firing rate modulation of all neurons averaged in ***A***. ***C***, PSTH of all left MTL single neurons with positive MIs. ***E***, PSTH of all left MTL single neurons with negative MIs. ***D***, ***F***, Heatmap of firing rate modulation of all left MTL neurons with increased (***D***) and decreased (***F***) firing in response to an IED. ***B***, ***D***, ***F***, Each row represents a neuron. Right, Scale of the normalized response. Color represents the proportional change relative to baseline (e.g., a value of 3 indicates the firing rate is 3 time higher than baseline). Neurons are sorted in descending order by the strength of their firing rate modulation. Horizontal line in (***A***,***C***,***E***) separates neurons from the hippocampus (top) from the amygdala (bottom). Red dashed line (***A***,***C***,***E***) indicates ± SE across neurons. Bin size of PSTH = 50 ms; bin size for heatmap = 25 ms. The time scale is different for heatmaps and PSTH.

To visualize the IED-related modulation in firing rate for each neuron, we plotted the normalized peristimulus time histograms (PSTHs) of the neurons as a heatmap (see, e.g., Fig. 3*B*). In these plots, each row represents a neuron, each column is a time bin (25 ms), and the color represents the change in firing rate from baseline (e.g., a value of 3 indicates the firing rate is 3 time higher than baseline). Neurons are sorted in descending order by the strength of their firing rate modulation.

#### Extracellular spike waveform analysis

**Figure 4.**
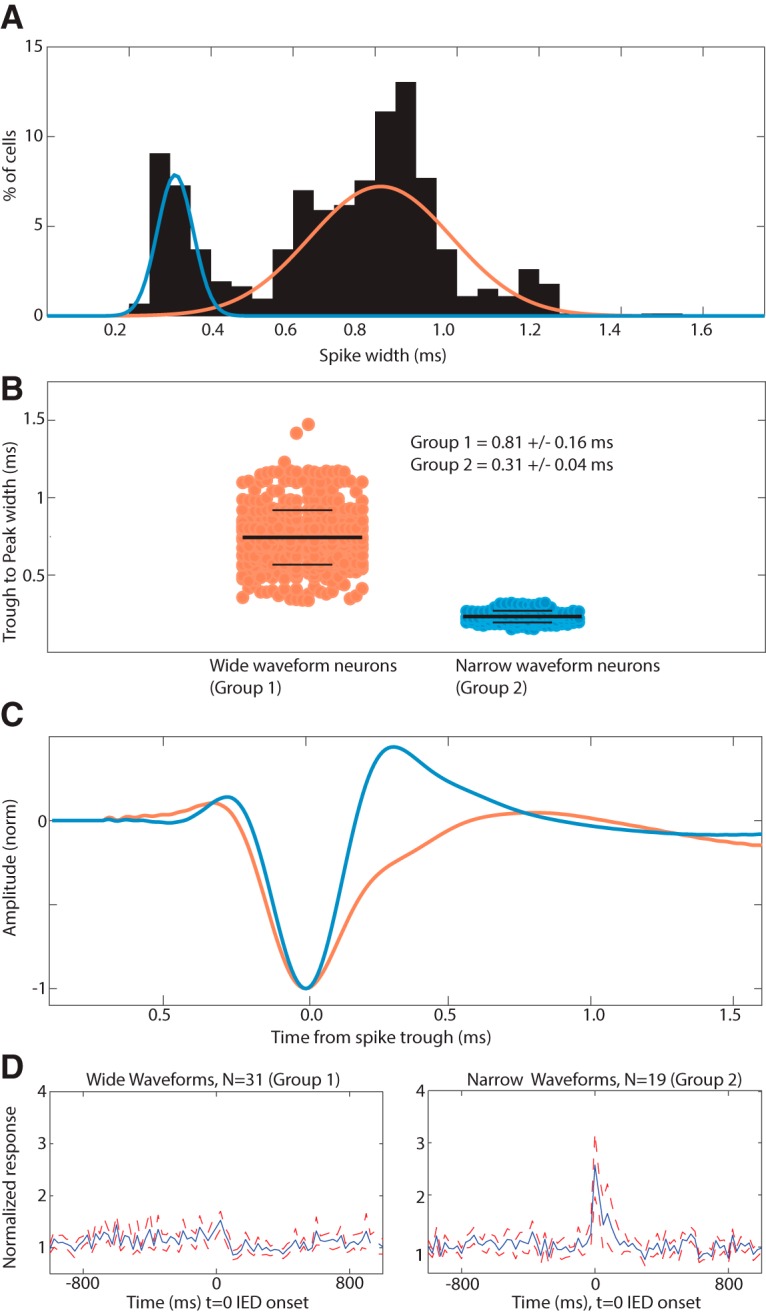
Cell type-specific modulation by IEDs. ***A***, Histogram of the distribution of spike widths of all single units analyzed. The two peaks indicate the presence of two distinct populations of neurons with the cutoff at ∼0.5 ms. ***B***, Distribution of spike widths of all the single units after splitting them into two groups: wide waveform cells (mean spike width of 0.81 ± 0.17) and narrow waveform cells (mean spike width of 0.31 ± 0.044 ms). ***C***, Average waveforms of the two groups shown in ***B***. ***D***, Group average PSTH of all modulated wide (left) and narrow width (right) single units across all the brain areas shows that neurons modulated with narrow waveforms on average increase their firing rate during IEDs, whereas the modulation of wide waveform neurons is more heterogeneous, resulting in little on-average modulation. Red dashed line indicates SE across neurons.

We used the extracellular waveform width to differentiate between different putative neuronal types (Barthó et al., 2004; Mitchell et al., 2007; Rutishauser et al., 2015; Takahashi et al., 2015). For each neuron, we calculated the trough-to-peak width of the average extracellular action potential. The trough was identified as the time point when the waveform was largest, and the peak is the first local maximum after the trough. The distribution of spike widths was bimodal (see Fig. 4*A*), as often observed in extracellular recordings. We classified cells as being narrow or wide spiking by performing *k*-means clustering on the trough-to-peak width of the spikes, selecting for two *k*-means groups.

#### Visualization

For plotting purposes, we binned each neuron's firing rate into 50 ms bins and averaged the firing rate over all neurons to calculate the PSTH (Koch, 1999).

#### Identification of selective cells

We characterized subsets of MTL cells according to their response to the visual category and novelty/familiarity of the presented visual stimuli as previously described. Briefly, a cell was characterized as visually selective (VS) if its response in a 1.5 s window starting 200 ms after stimulus onset was significantly modulated by the visual category of the stimulus (one-way ANOVA, *p* < 0.05) (Rutishauser et al., 2015; Faraut et al., 2018). A cell was classified as memory selective (MS) if its response in the same time window differed significantly as a function of whether the presented stimulus was novel or familiar (bootstrap test, *p* < 0.05) (Rutishauser et al., 2015; Faraut et al., 2018). Cells whose firing rate after stimulus onset across all trials differed significantly relative to baseline were classified as visually response (VR) cells. Some cells qualified as multiple types. Cells that were not classified as VS, MS, or VR cells were categorized as nonsignificant cells (NS).

#### Testing influence of IEDs on behavior

We used a GLM to test whether the likelihood that an image was correctly recognized or encoded varied as a function of whether an IED occurred within a given period of time in a given trial. For each trial of interest, we first determined the number of IEDs E (> = 0) that occurred within the time window of interest (a 3 s window, advanced from −3 s to 5 s relative to image onset) and whether the trial was correctly recognized or encoded C (0 or 1). We then fit the GLM, C ∼ 1 + E + (1|ID), where ID is a random factor that specifies the session ID. We fit this GLM to the data using a binomial response distribution function using *fitglme* in MATLAB.

To compare how well this model explained the data for different types of trials (recognition old, recognition new, learning trials), we used two approaches: (1) we compared the size of the weight for variable E between different models (each fit to one of the three trial types); and (2) we compared, for each model, whether it explained more variance compared with a null model. We compared the size of the estimated weight α_E_ of the model parameter E using its exponential, that is, exp(α_E_). This way, a weight of 0 is equivalent to an odds ratio of 1 (indicating no influence on the outcome). To estimate the significance of α_E_, we estimated the null distribution of α_E_ at every point of time using a permutation test (10,000 iterations). During every iteration, we first scrambled the order of the variable C (within each session), thereby preserving the average behavioral performance of each subject but destroying the trial-by-trial relationship. Using this null distribution, we then estimated the significance of α_E_. To estimate whether IEDs contributed significantly to explaining the data, we compared the fit to a null model without the model parameter E (null model specification, C ∼ 1 + (1|ID). We compared the full and null model using the log likelihood ratio. In addition to odds and log likelihood ratio, we confirmed the results also using the Akaike information criterion (AIC) to compare the two models.

#### Testing influence of IED-mediated neuronal modulation on behavior

We used a GLM to test whether the degree to which the activity of individual neurons was modulated by the occurrence of an IED was predictive of impairments of memory retrieval, here assessed by the confidence reported by the subject for each trial. The model we used was Conf ∼ 1 + A + E + (1|CellID) + (1|SessionID), where A is the number of spikes that a neuron fired during a given IED, E is the number of IEDs that occurred in this trial (here E ≥ 1), Conf is the confidence reported for this trial (high = 1 or low = 0), and CellID and SessionID are random factors to account for differences across neurons and patients. For this analysis, only neurons in the MTL significantly modulated by IEDs were included. Also, only trials during which at least 1 IED occurred were included (because the firing rate relative to an IED is undefined if there was no IED in a trial). The number of IEDs in each trial were counted in a 3 s time window, starting at −500 ms before stimulus onset (see Fig. 6*C*). To assess whether knowing the level of neuronal activity increased predictability, we compared this model with two different null models. Null model 1 was Conf ∼ 1 + E + (1|CellID) + (1|SessionID), which is identical to the full model, except the term A, thereby examining whether knowing the activity of neurons increases predictability beyond that already provided by the number of IEDs in a trial. Null model 2 was Conf ∼ 1 + A + (1|CellID) + (1|SessionID), thereby examining whether knowing the number of IEDs in addition to neural activity provides additional explanatory power. The number of spikes fired by a neuron A was counted in a window of size 100 ms. For the time course (see Fig. 6*D*), the position of this window was moved from −200 to 200 ms relative to IED onset (which was at *t* = 0) in steps of 5 ms. For the fixed time window analysis (see Fig. 6*C*), spikes were counted in the window −130 to 30 ms relative to IED onset (this window was picked because of the time course shown in Fig. 6*D*). For the model, confidence was computed as a binary index (high or low), and not a 6 point scale.

## Results

### Clinical characteristics of patients

The mean age of the patients was 49 ± 17.14 years (SD) (minimum 24, maximum 70). The most common etiology of the patients' epilepsy was medial temporal sclerosis. One patient had insular onset of unclear etiology, and 2 had bitemporal onset of their seizures. Resection was offered to 8 of these patients.

### Hippocampal IEDs preferentially modulate single neurons in the MTL

A total of 1871 hippocampal IEDs (Fig. 2*A*; 40% right hippocampal, 60% left hippocampal) were identified from 11 patients (Table 1). A total of 728 single units and 1871 IEDs were analyzed across 13 sessions. We first tested, for every neuron, whether its activity was significantly modulated by the occurrence of a hippocampal IED (two-tailed *t* test, *p* < 0.05, of firing rate quantified in bins of 50 ms before vs after the IED). An example of a significantly modulated unit in the right hippocampus is shown in Figure 2. We found that across all brain areas and patients, a small proportion of neurons (6.8%, *N* = 50 of 728, binomial, *p* = 0.016) were modulated by hippocampal IEDs. The extent of modulation differed significantly as a function of brain area (χ^2^ test of association between brain areas amygdala, hippocampus, and cortex and proportion of modulated cells: χ^2^_(2)_ = 9.6, *p* = 0.008; see also Table 2). *Post hoc* comparisons revealed that the proportion of neurons modulated in the hippocampus was significantly larger compared with both amygdala (χ^2^_(1)_ = 6.90, *p* = 0.009) and cortex (χ^2^_(1)_ = 6.94, *p* = 0.008). For all recorded MTL neurons, a significant proportion were modulated (33 of 416, binomial, *p* = 0.007). Comparing between different hemispheres, modulation was significantly higher for neurons recorded from the right compared with the left hippocampus: χ^2^_(1)_ = 5.93, *p* = 0.015; 20% (*N* = 12 of 58) versus 7.6% (*N* = 8 of 105), respectively. The proportion of modulated cells was not significantly different from that expected by chance in the amygdala (right: 5.21%, *N* = 6 of 115, binomial, *p* = 0.52; left: 5%, *N* = 7 of 138, binomial, *p* = 0.54) and did not differ significantly between the left versus right side (χ^2^_(1)_ = 0.001, *p* = 0.97). In the MTL, the majority of modulated neurons (75.75%, *n* = 25 of 33) were contralateral to the seizure onset zone. Additionally, a majority of the right temporal lobe neurons modulated by IEDs (88.8%, *n* = 16 of 18) were contralateral to a left hemispheric seizure onset zone. We next tested whether neurons recorded in the cortex are modulated by hippocampal IEDs. Across all cortical areas recorded from, a relatively small and not significant proportion of cells showed such remote modulation (17 of 312, 5.4%; Table 2). This was also true when considering brain areas individually, with no significant differences between areas in the propensity to be modulated by hippocampal IEDs (χ^2^ test of association between brain areas pre-SMA, ACC, and orbitofrontal cortex vs proportion of modulated cells: χ^2^_(2)_ = 1.09, *p* = 0.58). Together, this shows that the neurons that were most modulated by hippocampal IEDs were those recorded in the hippocampus, with no significant modulation of neurons in the other recorded brain areas.

**Table 2.** Number and percentage of modulated single units for all the sessions during the new-old task

Brain area	No. of modulated cells/total cells	Percentage of modulated cells (%)
Left anterior cingulate	2/20	10
Left pre-SMA	6/107	5.6
Left amygdala	7/138	5
Left hippocampus	8/105	7.6
Left orbitofrontal	1/19	5
Right anterior cingulate	2/50	4
Right pre-SMA	3/85	3.52
Right amygdala	6/115	5.21
Right hippocampus	12/58*	20
Right orbitofrontal	3/31	9.6
Medial bitemporal	33/418*	8.0

*p < 0.05, binomial test versus chance of proportion of identified neurons.

In the MTL, cells can be characterized into different functional categories based on their response to the visual stimulus shown during the recognition memory task (Table 3) (Rutishauser et al., 2015; Faraut et al., 2018). Here, as done previously, we characterized MTL cells based on their response pattern as either VS (meaning their response differs as a function of the category of the visual image), MS (response differs according to whether the image is new or old), or neither. We then evaluated separately for each of the groups of cells what proportion was modulated by IEDs. While the proportions varied somewhat between the different cell types, there was no significant difference between the different functional cell types in their propensity of being modulated by IEDs (χ^2^ test of association between brain cell types MS, VS, and other: χ^2^_(2)_ = 1.00, *p* = 0.61; Table 3). This shows that IEDs tend to modulate differentially tuned cells indiscriminately.

**Table 3.** Number of modulated single units based on the characteristic type

Brain area	No. of modulated cells/total cells			
	MS	VS	VR	NS
Left amygdala	0/11	1/24	2/38	4/65
Left hippocampus	1/8	1/23	3/32	3/42
Right amygdala	1/7	1/23	0/27	4/58
Right hippocampus	1/3	1/6	3/22	7/27
Medial temporal left + right (%)	10	5	6	9

### Temporal pattern of modulation by IEDs

We next compared the pattern of modulation across all IED-modulated neurons. For this, we determined for each modulated neuron whether the modulation was positive or negative as indicated by the sign of the MI, which compares the firing rate of neurons between a 50-ms-wide window before versus after the onset of an IED (see Materials and Methods). If the MI was negative, it indicated an IED-modulated decrease in firing rate comparing before versus after IED onset. In contrast, if the MI was positive, this indicated an IED-modulated increase in firing rate relative to the firing rate immediately before IED onset. Across all brain areas, 35 modulated single units had a positive MI (mean SD, 0.43 ± 0.17), while 15 had a negative MI (−0.18 ± 0.70). In the right MTL, the MI of all IED modulated single units was positive (mean ± SEM, 0.40 ± 0.03, Cohen's *d* score = 0.24 ± 0.02). The left temporal lobe did not show this preferential distribution of MI; with 8 units being positive (0.42 ± 0.05, Cohen's *d* score = 0.23 ± 0.04) and 7 being negative (0.54 ± 0.09, Cohen's *d* score = −0.30 ± 0.06). The negative or positive MI values can result from several different patterns, including changes only before or after IED onset but also a more complex pattern, such as inhibition of firing after relative to before IED onset. To further investigate these differences, we plotted a group PSTH centered around the IED separately for units with positive and negative MI. This revealed that the *n* = 18 positively modulated cells (none negative) in the right temporal lobe transiently increased their firing rate in the 50 ms window following IED onset at *t* = 0, with no modulation extending beyond ∼100 ms after IED onset (on average; see Fig. 3*A*,*B*). In the left temporal lobe (Fig. 3*C–F*), on the other hand, there were two temporal patterns of modulation: while both groups exhibited (on average) an increase in firing rates due to IEDs, this increase either followed (Fig. 3*C*) or preceded (Fig. 3*E*) the IED onset by ∼100 ms. The neurons with negative MI, on the other hand, exhibited little modulation on average, indicating that such modulation is either heterogeneous or weak (Fig. 3*E*,*F*).

### IEDs preferentially increase firing of putative inhibitory neurons in the right temporal lobe

We next asked whether different electrophysiological types of cells are differentially affected by IEDs. To achieve this, we characterized the neurons that were significantly modulated by IEDs based on the trough to the peak width of their extracellular waveform (i.e., the action potential). Neurons with narrow action potentials are thought to be GABAergic interneurons, whereas those with wider action potential (>0.5 ms) are thought to be excitatory neurons (Barthó et al., 2004; Mitchell et al., 2007; Rutishauser et al., 2015; Takahashi et al., 2015).

As expected (Rutishauser et al., 2015; Fu et al., 2019), pooling neurons across all the brain areas we studied, the distribution of neurons was bimodal with the cutoff between the two groups equal to 0.52 ms (Fig. 4*A*,*B*). The majority of cells had wide action potentials (71%, *n* = 571), compared with narrow waveform neurons (21.5%, *n* = 157) (Table 4). Figure 4*C* shows the average waveform of the two groups. This is compatible with earlier work (Rutishauser et al., 2015) and indicates that the majority of neurons recorded are putatively excitatory pyramidal cells. We next tested separately for narrow and wide waveform neurons whether their activity was modulated by IEDs. This revealed that neurons with narrow waveforms were significantly more likely to be modulated by IEDs compared with neurons with wide waveforms (19 of 157 vs 31 of 571; 12.1% vs 5.4%; significantly different, *p* = 0.0034, χ^2^ test). In addition, the modulated units with narrow waveforms, which are putative interneurons, were significantly more likely to increase rather than decrease their firing in response to the IEDs (14 of 19 increase vs 5 of 19 decrease; *p* = 0.0035, χ^2^ test). This was also true for wide-waveform neurons (Table 5). In conclusion, IEDs were more likely to modulate narrow-waveform neurons, and this modulation was more likely to be an increase rather than decrease of firing rate (Fig. 4*D*).

**Table 4.** Number of IED-modulated narrow and wide waveform cells across all brain areas

Type	IED modulated	IED nonmodulated	Total
Narrow waveforms	19	138	157
Wide waveforms	31	540	571

**Table 5.** Number of modulated single units in the entire brain based on their firing pattern

Type of modulation	Narrow waveforms	Wide waveforms	Total
Increased firing of units	14*	21*	35
Decreased firing of units	5*	10*	15
Total	19	31	50

*p < 0.05, binomial test versus chance of proportion of identified neurons.

We next repeated the above analysis for only MTL neurons (above, all neurons across all brain areas were pooled). Most MTL neurons had wide waveforms (81%, *N* = 339 of 418), of which only 6.5% (*n* = 22) were modulated by IEDs. Of the narrow waveform neurons (19%, *N* = 79 of 418), 13.9% (11 of 79) were modulated by IEDs (Table 6), a proportion significantly larger than that for wide-waveform neurons (*p* = 4.5e-4, χ^2^ test). We did not find a significant difference in the proportion of narrow-waveform neurons between right and left temporal lobes (Table 7). The neurons modulated by IEDs in the MTL contralateral to the seizure focus showed a slightly higher proportion of narrow waveforms (81%, *N* = 9 of 11), compared with wide-waveform neurons (73%, *N* = 16 of 22), and both types of cells were equally likely to increase their firing during IEDs. This result shows cell type specificity of modulation by IEDs.

**Table 6.** Number of modulated single units in the right and left MTL (hippocampus and amygdala) based on their firing pattern

Type of modulation	Narrow waveforms	Wide waveforms
Increased firing of units	7*	19*
Decreased firing of units	4	3
Total	11	22

*p < 0.05, binomial test versus chance of proportion of identified neurons.

**Table 7.** Number of modulated single units in the right and left MTL (hippocampus and amygdala) based on their firing pattern

Area	Narrow waveforms (modulated by IED/total)	Wide waveforms (modulated by IED/total)	Total
Right temporal	6/40*	12/138*	178
Left temporal	5/39*	10/201	240

*p < 0.05, binomial test versus chance of proportion of identified neurons.

### IEDs that appear within 2 s of image presentation predict disruption of retrieval of old memories

We next tested whether the occurrence of an IED had an effect on behavior by testing whether accuracy in the recognition memory task was affected by whether an IED occurred or not in a given trial. We were particularly interested in the temporal sensitivity of this effect and thus evaluated this effect separately for different points of time between IED onset and stimulus onset. For this, we used GLM models to assess whether the probability of correctly retrieving an image (or later remembering for encoding trials) was correlated with the presence of IEDs (see Materials and Methods). We fit one model each to all old trials during recognition, all new trials during recognition, and all learning trials. We then compared these models with a null model that was equivalent, except for the IED variable, which was removed. We quantified the significance of these model comparisons using both the log likelihood ratio and AIC.

**Figure 5.**
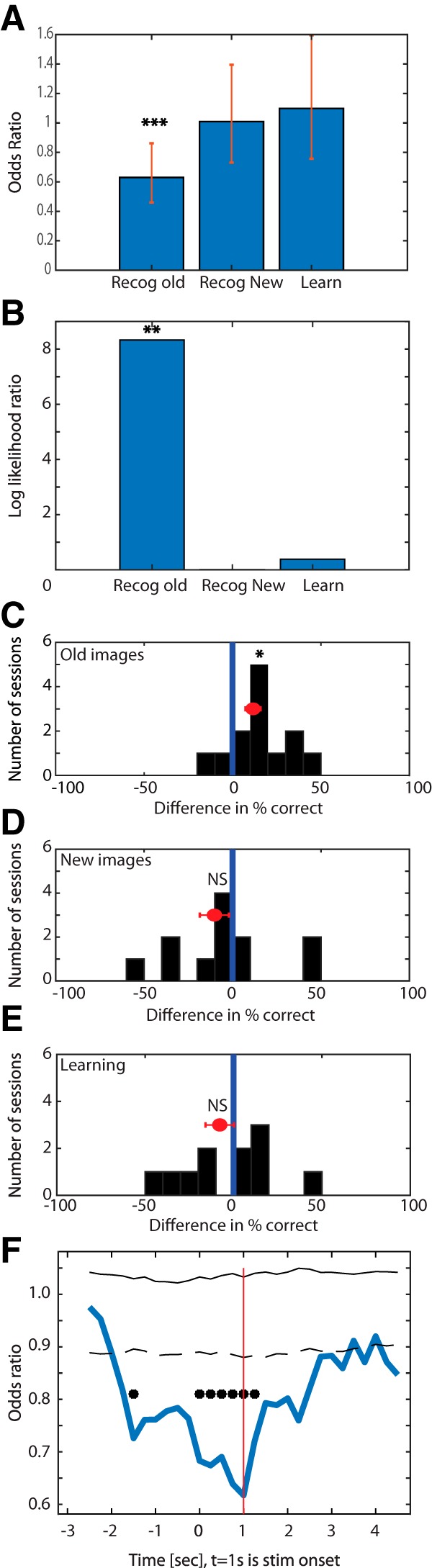
Behavioral effects of IEDs during different task phases. ***A***, Results of different GLM models to assess the impact of IEDs on behavior during different types of trials. During the recognition phase of the task, the presence of IEDs during a given trial significantly reduced the likelihood that an image will be remembered correctly. In contrast, there was no significant change in the likelihood of a new image being recognized as such during recognition or in the likelihood that a new image during learning (right) was later remembered correctly. Bars represent an independent GLM model fit to the indicated subset of trials. Error bars indicate CIs (odds ratio 0.63; ***p = 0.004). ***B***, Model comparison versus a null model without access to when IEDs occurred. Compared with the null model, the model that takes into account when IEDs occurred was significantly more likely given the behavioral data (**p = 0.01) for recognition old trials. No multiple comparison correction was performed as each bar is the result of a different model on an independent subset of trials. ***C***, ***D***, Difference in behavioral performance for each subject between trials with none versus at least 1 IED. This revealed a significant difference in the proportion of correctly remembered old images (***C***, shift to the right ***C***, paired *t* test, *p* = 0.02), with no difference in the proportion of correctly identified new trials (***D***). ***E***, Same as ***C*** and ***D***, but for learning trials. Shown is the difference in the proportion of later correctly remembered learning trials between trials in which there was no versus at least 1 IED. There was no significant difference (*p < 0.05). ***F***, Time course (blue line) of the odds ratio for the model shown in ***A*** for recognition old trials. Stimulus onset is at *t* = 1 s (red line). The largest effect of IEDs was around stimulus onset. Bin size = 3000 ms (plotted points are the center of this bin). Black line indicates the null model. Dashed line indicates SE. *p < 0.01, after correcting for multiple comparisons with FDR across all time points shown. Null distribution was established using a bootstrap, scrambling the order of trials within each subject, repeated 10,000 times for each time point. NS = not significant.

We found that, when IEDs occurred during a retrieval trial in which an old image was shown, the old images were more likely to be forgotten (i.e., subjects were more likely to say it was new, thus a false negative; odds ratio = 0.63, *p* = 0.004; Fig. 5*A*, left). A model comparison revealed that the model with access to IEDs was significantly more likely than a null model without access to this variable (Fig. 5*B*, left; log likelihood ratio = 8.32, *p* = 0.01; also confirmed using AIC = 747.98 < 752.57). Fitting the same model to new trials during recognition revealed that the probability of correctly identifying a new trial (i.e., a true negative) was not significantly correlated with the presence or absence of IEDs (Fig. 5*A*, middle; odds ratio = 1, *p* = 0.96). This impression was confirmed by a model comparison with a null model without access to IEDs, which showed no significant difference (log likelihood ratio = 0.003, *p* = 0.96; AIC = 667.91 > 665.91). Last, we tested whether the presence or absence of IEDs affected the probability that a memory was successfully formed during encoding. To evaluate this, we tested whether the probability that a new image shown during the learning phase would later be correctly recognized as old was influenced by the presence or absence of an IED during encoding of that particular image. We found no significant relationship (Fig. 5*A*, right; odds ratio = 1.1, *p* = 0.64; model comparison shown in Fig. 5*B*, right; log likelihood ratio = 0.25, *p* = 0.62, AIC = 576.34 > 574.59). This thus indicates that the presence of IEDs did not disrupt the encoding process.

To provide further intuition into the result of these model comparisons, we also visualized the difference in behavioral performance between trials with and without IEDs, separately for the three different trial types investigated above (Fig. 5*C–E*). However, this is for illustration only because this univariate interpretation does not account for factors such as repeated measures of multiple neurons in the same subject and between-subject variability in firing rates that the multivariate analysis performed above using GLMs takes into account. Nevertheless, these univariate analyses confirmed the impression given by the GLMs: performance differed significantly between trials with and without IEDs for recognition old trials (Fig. 5*C*; paired *t* test, *p* = 0.02) but not for recognition new trials (Fig. 5*D*; paired *t* test, *p* = 0.26) and learning trials (Fig. 5*E*; paired *t* test, *p* = 0.36).

We next tested whether the effect of the occurrence of IEDs during the retrieval of old images varied as a function of time. For this, we evaluated above model (on recognition old trials) separately for different points of time relative to stimulus onset, counting only IEDs that occurred within a window of ±1.5 s around the center of the bin (3 s time window; plotted point is center of window in Fig. 5*F*). This revealed that the effect of the IED on correct retrieval of an old image was strongest if the IED occurred approximately at stimulus onset (Fig. 5*F*). IEDs that appeared up to 2 s before stimulus onset also significantly impaired retrieval. In contrast, as expected, IEDs that occur >1.5 s after stimulus onset did not influence retrieval (Fig. 5*F*). Together, this correlation between behavior and IED timing shows high temporal specificity of IEDs, with the strongest effect observed if an IED occurred simultaneously with stimulus onset.

### Modulation of neuronal activity by IEDs predicts reduced confidence

**Figure 6.**
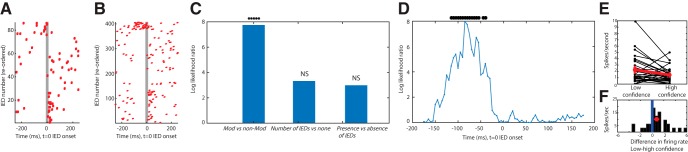
Extent of modulation of the activity of individual neurons by IEDs predicts reduction in behaviorally declared memory retrieval strength (confidence). ***A***, ***B***, Raster plots of two example neurons that are modulated by IEDs. Each row represents a different IED (*t* = 0 is onset of the IED). Rasters are rank ordered by the number of spikes fired in a window −100 to 0 ms relative to IED onset. Note the substantial trial-by-trial variability in modulation. ***A***, Same unit as shown in Figure 2*A–C*. ***B***, Example unit from p49. ***C***, Model comparisons between different models that predict the confidence (high or low) of a recognition trial as a function of the firing rate of recorded neurons and the number of IEDs observed in a given trial. The model with access to both neuronal activity around IEDs (time window −130 to −30 ms relative to IED onset) and the number of IEDs performs significantly better than a model with only access to the number of IEDs (left, *p* = 0.005; middle, *p* = 0.07; right, *p* = 0.08). ***D***, Time course of the model comparison shown on the left in ***C***, quantified by the log likelihood ratio between the full model and the model with only access to the number of IEDs (bin size = 100 ms, step size = 5 ms; plotted data point is center of the bin). The firing rate of neurons was most informative about whether a trial would be rated as high or low confidence ∼100 ms before IED onset (*t* = 0). *p < 0.05 (uncorrected). ***E***, Neuron-by-neuron comparison of mean firing rate at the time of IED occurrence, shown separately for low and high confidence trials. This shows that the greater the increase in firing rate, the lower the confidence (left vs right, Kolmogorov-Smirnov test, *p* = 0.03). Each line is a neuron. ***F***, Summary of ***E***. Histogram of difference in the firing rate of neurons around IEDs between low and high confidence trials for all the neurons in the MTL modulated by IEDs. This shows that the difference was shifted to the right for low confidence trials. NS = not significant.

The above results reveal a relationship between the occurrence of IEDs and behavior as well as modulation of the activity of individual neurons. However, it remains unclear whether the two phenomena are related. Examining individual neurons that were significantly modulated by IEDs on average revealed substantial IED-by-IED variability in this modulation (Fig. 6*A*,*B*). We thus hypothesized that the variable degree of modulation of neurons by a given IED would provide a tool to examine correlations of IED-modulated neuronal modulation with behavior. Here, we used the subjective confidence reported by the subject (the declarative aspect of this recognition memory task) as a sensitive behavioral readout of the retrieval process. We used a GLM to assess the extent to which the subjective confidence provided by a patient for a given recognition trial (regardless of whether it was new or old) was related to the degree by which neurons changed their activity around the onset of IEDs. This population-level model consisted of the pooled activity of all IED-modulated neurons in the MTL and all trials in which at least 1 IED occurred (see Materials and Methods). We first compared the full GLM model with access to both the firing rate of neurons around an IED and the number of IEDs that occurred (see Materials and Methods) with one that only had access to the number of IEDs. This revealed that the full model with access to neuronal activity explained significantly more variance in the confidence judgments provided by the subjects (Fig. 6*C*, left; *p* = 0.005; note the effect size of approximately an eightfold increase). In contrast, comparing a model that has only access to the number of IEDs with one that has no such access was not able to explain significantly more variance than the null model (Fig. 6*C*, middle; *p* = 0.07). Also, comparing the full model with one where only the number of IED term was dropped (providing the model with only access to neuronal firing rates) also did not reveal a significant drop in ability to explain variance in confidence judgments (Fig. 6*C*, right; *p* = 0.08). Together, these model comparisons indicate that firing rate around IEDs was the best predictor. We next examined the full model more closely. The weight of the firing rate parameter was significantly different from 0 and negative (−0.046, *p* = 0.0053, CI −0.078 to −0.014). Since the coding for confidence was such that a higher value equals higher confidence, this indicates that higher firing rates of neurons around IEDs lower recognition confidence. We confirmed this impression by performing a univariate analysis for visualization only (for statistics, see Fig. 6*E*,*F*, legend).

Last, we tested whether the effect on confidence of recognition by the modulation of IEDs varied as a function of time. For this, we evaluated the same full GLM model as discussed above, but at different time points relative to IED onset (bin size 100 ms, step size 5 ms). This revealed that the effect of modulation of a single-neuron activity on confidence of recognition was strongest for spikes occurring in a window from −130 to 30 ms before the onset of IEDs (Fig. 6*D*). This shows that the effect of IED-modulated firing rate changes on memory retrieval (as assessed by confidence) has high temporal specificity, with respect to onset of the IED, with the strongest effect observed before onset on intracranial EEG.

## Discussion

We found that hippocampal IEDs are associated with a decrease in the likelihood of correctly retrieving an existing memory. In contrast, we found no effect on the encoding of new memories, a finding that is different from a previous studies that suggested that IEDs impair encoding of new memories (Horak et al., 2017; Ung et al., 2017). Note, however, that we used a hippocampal-dependent recognition memory task whereas this previous work used a delayed free recall task. It is thus possible that selective impairment of retrieval is specific to long-term memory. We also provide the first single-unit analysis of firing modulation by IEDs during a recognition memory task, which shows that neurons are modulated during active performance of a task. In contrast, previous work has evaluated modulation of IEDs during rest (Creutzfeldt et al., 1993; Keller et al., 2010; Alvarado-Rojas et al., 2013). IEDs can differ markedly between rest and active task performance (J.Y. Matsumoto et al., 2013), making it important to study IED-related modulation during performance of a task. We also found that modulation of single-neuron activity by IEDs was more pronounced in the right MTL. Additionally, a greater proportion of right medial temporal neurons modulated by IEDs were contralateral to a left hemispheric seizure onset zone. It is possible that these areas were healthier, hence more likely to respond to IEDs.

The occurrence of IEDs has been shown to predict decreases in performance during encoding and retrieval in a free-recall task (Ung et al., 2017). Similarly, a second study found that increased rates of IEDs in neocortical and left hemispheric areas were correlated with impaired encoding and recall to a greater extent (Horak et al., 2017) compared with right hemispheric IEDs. We found that hippocampal IEDs impacted recognition but not encoding. The odds ratio we observed was similar to that obtained in the previous study (Horak et al., 2017). Also, in our experiment, we were able to differentiate between effects related to the presentation of novel (“new”) images, the effects of task demands (learning vs retrieval), and effects related to specific images themselves. This is because we repeated the same images that were new during learning during retrieval, intermixed again with new images. We found that the behavioral effects of IEDs were specific to old images during recognition, but not the recognition of new images during recognition or their encoding during learning.

In humans, single-neuron studies have revealed that a subset of ∼30% of neurons modulate their firing transiently prior or during an IED (Alarcón et al., 2012; Alvarado-Rojas et al., 2013). The modulation of single-unit firing at the start of the IED is thought to be due to paroxysmal depolarization shift. The initial depolarization phase of an IED is thought to represent glutamate receptor, mainly AMPA- and NMDA- mediated calcium conductance (Traub and Wong, 1982; Trevelyan et al., 2006). The increase in neuronal firing around the IED is followed by decrease in firing in the post-IED period (Wyler et al., 1982; Keller et al., 2010; Alvarado-Rojas et al., 2013). The ensuing hyperpolarization phase is thought to represent GABA-mediated inhibition (Cohen et al., 2002), and is also accompanied by decreased rate of neuronal firing (Altafullah et al., 1986; Ulbert et al., 2004; Alvarado-Rojas et al., 2013). This period of suppression is longer and has been shown to be accompanied by large current sources in middle cortical layers (Trevelyan et al., 2007). The modulation of single-unit firing in our study showed significant changes in firing compared with the baseline firing rate in the 50 ms before the onset of the IED, indicating that the effects of IEDs on the firing rate of neurons precedes the time when the IED is visible at the field potential level. Our MI is a more sensitive measure of IED-induced changes in firing rates than simply comparing changes in single-unit firing probability (Alvarado-Rojas et al., 2013), since it incorporates information about baseline firing rates immediately before IED onset.

The proportion of neurons modulated in our study were smaller than in previous studies. In contrast to the 20% we found to be modulated in the right MTL (hippocampus and amygdala), earlier studies found that during sleep 30% of hippocampal neurons (Alvarado-Rojas et al., 2013) and during quiet wakefulness 48% of all neurons (Keller et al., 2010) are modulated by IEDs. The IED rates in our study and these previous studies are similar (0.086/s vs 0.057/s) (Keller et al., 2010). However, in general, cognitive load is believed to lower IED rates (Aarts et al., 1984; J. Y. Matsumoto et al., 2013), leaving open the possibility that at rest the IED rates in our patient would have been higher. The lower modulation rates in our versus previous studies supports the hypothesis that performance of a recognition-memory task lowers the effect of IED on single-neuron activity. If so, this would indicate that engagement of neurons by IEDs can be changed flexibly based on task demands, a feature that could possibly be used for new strategies to reduce the impact of IEDs.

We found that the occurrence of IEDs during retrieval, but not encoding, was predictive of impaired performance. This disruption was temporally specific. This is compatible with earlier work, which showed that hippocampal IEDs that occurred during retrieval, but not during the maintenance phase of a Sternberg working memory task, predicted a decrease in response accuracy (Kleen et al., 2013). Prior work in children with a short-term memory test, presented as an engaging television game, found that right-sided discharges caused impairment of the spatial version of the task, while left-sided with impairments on the verbal version (Binnie et al., 1987). These effects were also temporally specific. Thus, the timing of IEDs relative to ongoing task events is critical to their behavioral impact, arguing for a highly specific and transient mechanism rather than more general and long-lasting impairment.

Linking the neuronal and behavioral effects of IEDs, we found that the degree to which single-neuron activity in the MTL was modified by IEDs was predictive of decreases in retrieval confidence. The timing of this was specific, with the most predictive power being the activity of neurons during the period of −130–30 ms before the onset of the marked onset time of the IED. An IED is thought to represent the extracellular correlate of the synchronous and excessive discharge of a group of neurons, and is believed to be preceded by a paroxysmal depolarizing shift (H. Matsumoto and Ajmonemarsan, 1964; Dichter and Spencer, 1969; Wong and Traub, 1983; de Curtis et al., 1999; de Curtis and Avanzini, 2001). Thus, it would be expected that changes in the activity of individual neurons would be observed before the onset of the IED itself and that these changes would be most reflective of synchronous synaptic input. Our finding that activity changes shortly before IED onset are most predictive of changes in retrieval confidence is compatible with this interpretation. Together, this result reveals a first direct link between the degree by which an individual IED modulates the activity of neurons in the MTL and a behaviorally measured impairment in declarative memory, here assessed by confidence.

To put our findings in perspective, consider that there are ∼48 and 12 million neurons in each hippocampus and amydala, respectively (Simic et al., 1997; Schumann and Amaral, 2005). Our finding that on average 8% of neurons were significantly modulated thus implies that ∼9 million neurons per hemisphere changed their firing rate due to an IED. This large-scale modulation likely explains our ability to correlate the modulation strength of individual neurons around an IED with behavior.

Our results call to attention the phenomenon of transient cognitive impairment, which is believed to be related to IEDs (Aarts et al., 1984; Binnie, 2003). The main feature of transient cognitive impairment is the time-locked nature of the IED with the disruption. To our knowledge, ours is the first study to investigate a putative mechanism for transient cognitive impairment. The increased firing of a greater proportion of inhibitory interneurons compared with the excitatory neurons, especially in the right MTL, could signify a possible mechanistic link to the behavior we see when retrieving old images and the disruption of confidence of recognition (i.e., retrieving an existing memory). Mechanistically, a transient and disproportionate increase in inhibitory interneuron firing could block local network and intra-areal transmission of information within the MTL, therefore impacting recall of learned information.

In conclusion, this study provides critical new insights into the mechanisms by which IEDs impair human cognition. The task used here is a recognition memory task with the explicit declarative component of confidence ratings, which are a highly sensitive behavioral measure of memory strength (Rutishauser et al., 2006b; Squire et al., 2007). In this task, hippocampal IEDs preferentially and transiently impaired retrieval of familiar images, preferentially modulated the activity of putative inhibitory neurons in the MTL, and the engagement of neurons shortly before IED onset predicted reductions of retrieval confidence. More broadly, this study demonstrates that examining the effects of IEDs at the single-neuron level provides a way to start understanding why and how specifically IEDs impair human cognition.
